# Supplementary material for: Behavior change and infection induced immunity led to the decline of the 2022 Mpox outbreak in Berlin
Source: Commun Med (Lond). 2026 Jan 6;6:81. doi: 10.1038/s43856-025-01340-5 (PMC12873328; doi:10.1038/s43856-025-01340-5)
Supplement: Supplementary file 2 — Supplemental Material [file 43856_2025_1340_MOESM2_ESM.pdf]

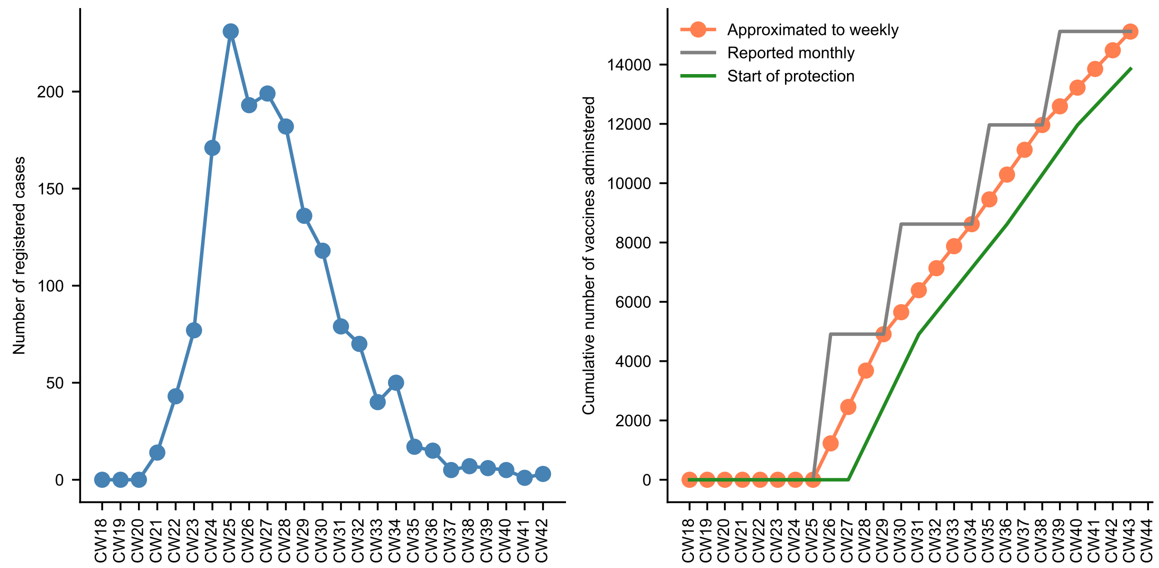

Supplementary Figure 1: Registered cases per calender week (CW) and cumulative number of vaccinations administered per month, extrapolated to calender weeks. We use the reported number of new infections per calendar week from 2022-05-11 to 2022-10-18, adding two weeks at the beginning with no reported infections, giving a period of 26 weeks. We took the number of first vaccine shots from the vaccination timeline and divided it by the number of calendar weeks per month to get the number of first shots per calendar week. We assume that the protection from the vaccine and its behavioral effects start 14 days after administration [1]. We then shift the vaccination timeline by two weeks, assuming the vaccination effects happen immediately.

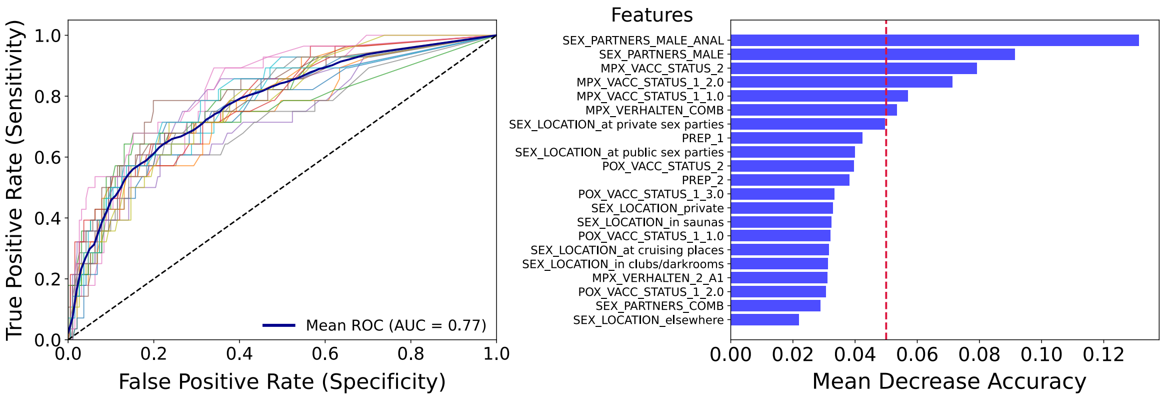

Supplementary Figure 2: Random Forest model performance and variable ranking to identify categories that explain positive seroprevalence from surveydata [2]. Left: Bootstrap-based ROC curves (colored lines), and mean ROC curve (dark blue). The diagonal line represents an AUC of 0.5 (random classification). Right: Variable importance plot. A higher mean decrease in accuracy indicates greater importance in the model. Variables with a mean decrease in accuracy above 5% (vertical line) were selected. Both linear models (e.g., linear regression) and non-linear approaches (e.g., SVM, Random Forest) were used and compared to the dataset. The dataset was split into distinct training and test sets, and cross-validation was used to improve the model reliability. Random Forest was chosen as the final model based on performance metrics (left), with an accuracy of approximately 86.3 %. Model provided feature importance ranking was used to identify and select key variables (right). Selected variables including the number of anal male sexual partners, mpox vaccination status and mpox-related behavioral changes were used for the population-based network contact model.

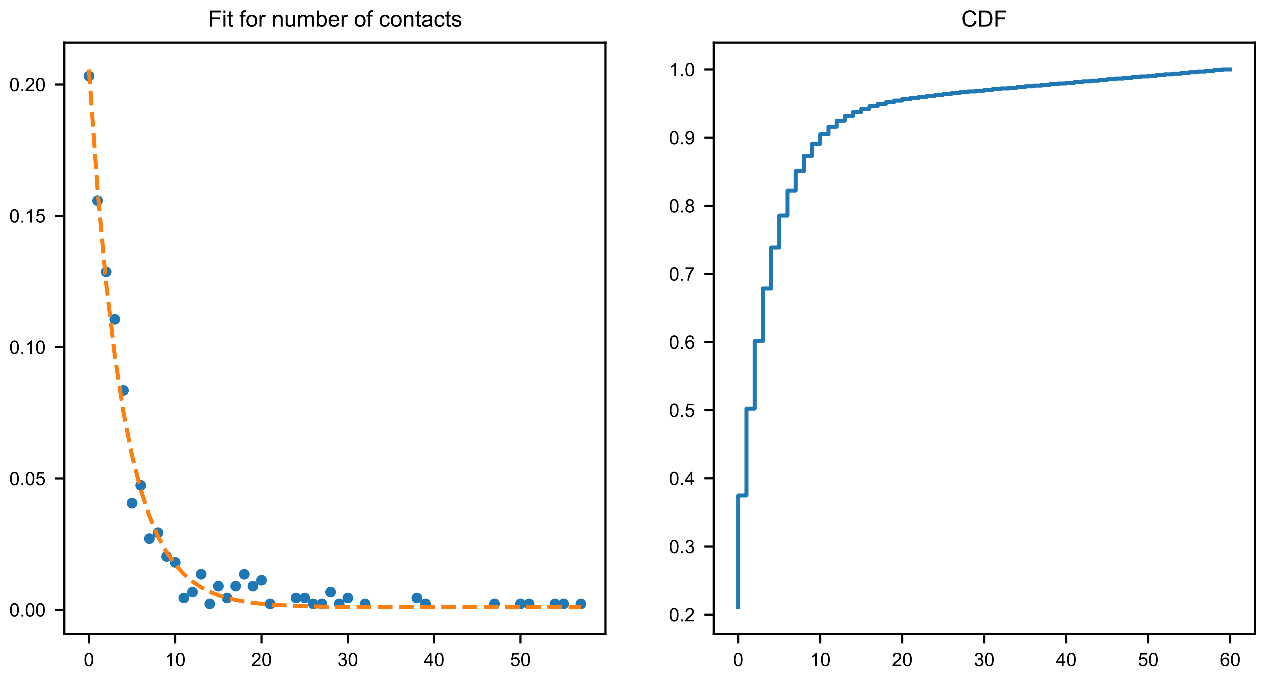

Supplementary Figure 3: Distribution and fit of contacts in the vaccinated subgroup. CDF of fit on the right.

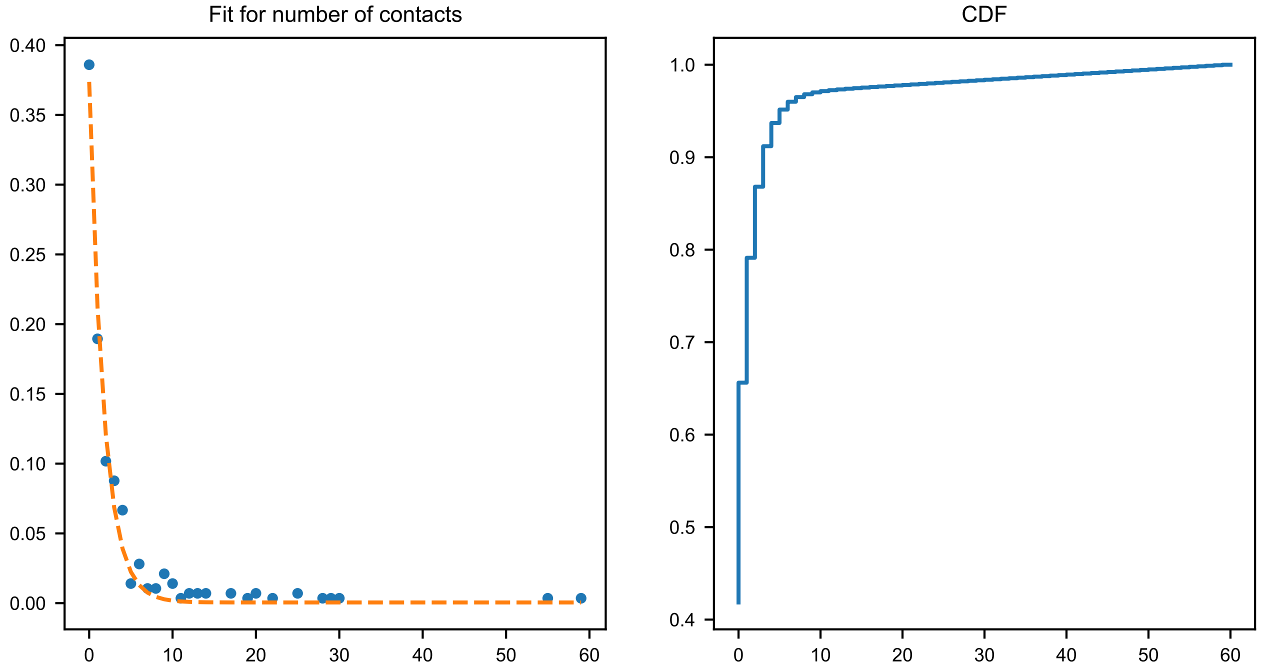

Supplementary Figure 4: Distribution and fit of contacts in the non-vaccinated subgroup. CDF of fit on the right.

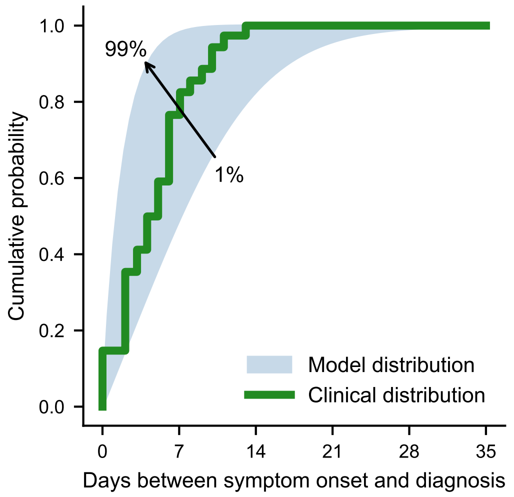

Supplementary Figure 5: Cumulative distribution function (CDF) of diagnostic delay for values of p_{diag} between 1-99%. Compared with [3]. In each infectious compartment I_j ($j \in \{1,2,3,4\}$ the agent either moves to the next compartment I_{j+1} or gets diagnosed. In the last infectious compartment I_5 the agent either gets diagnosed or recovers. Rates for transition between these compartments are collected in a rate matrix $\boldsymbol{Q}$. The transition probabilities at time t are then given as $\boldsymbol{P} = \exp(\boldsymbol{t}Q)$. The diagnosis probability is calculated by looking at the limit $\lim_{t\to\infty}(\boldsymbol{\pi}(\boldsymbol{0})\boldsymbol{P}(t))_D$, where $\boldsymbol{\pi}(\boldsymbol{0})$ is the initial distribution with all agents in the first infectious compartment.

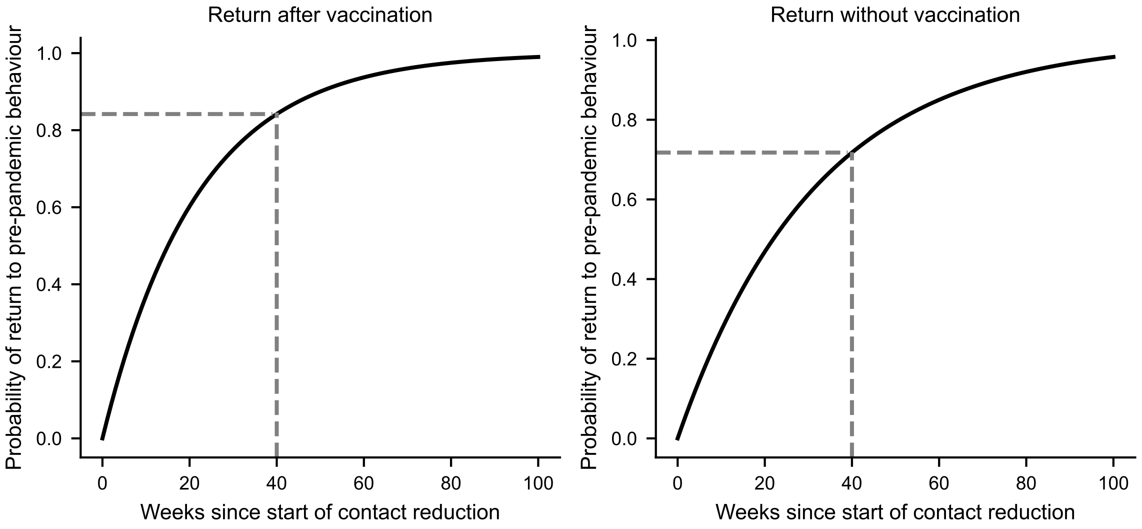

Supplementary Figure 6: CDF of time until return to pre-pandemic behaviour. The grey dotted lines represent the observed data, black line is the model fit.

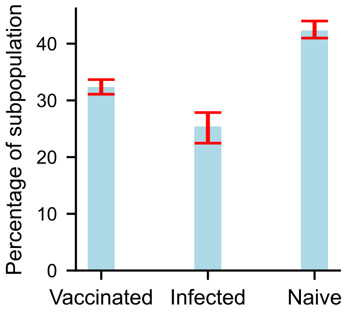

Supplementary Figure 7: Immune status of agents with more than 5 contacts per year at the end of the simulation.

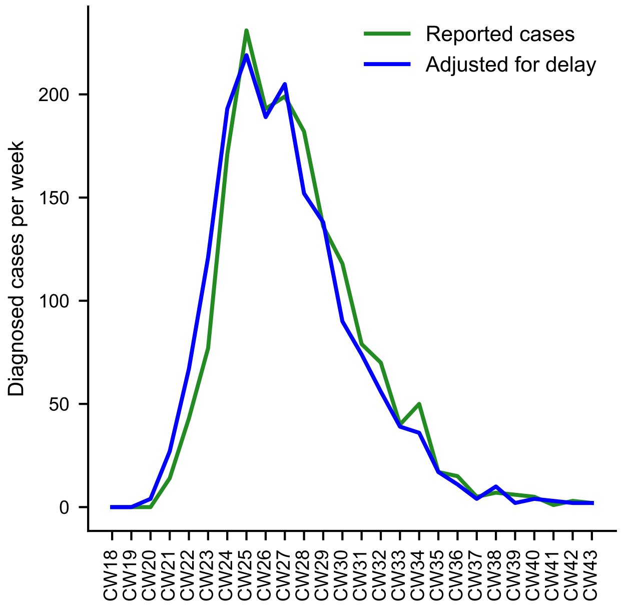

Supplementary Figure 8: Reporting delay adjusted cases compared to reported cases. Artificial reporting delay was created by adding exponentially distributed noise to the reporting dates.

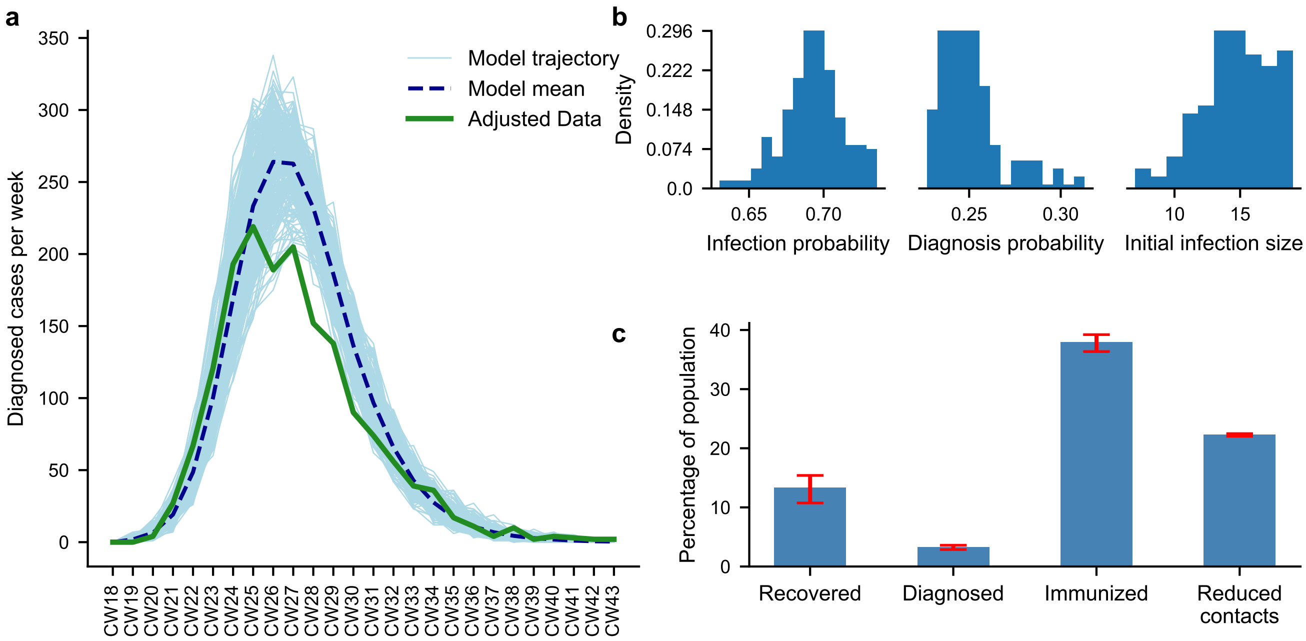

Supplementary Figure 9: **Overview of epidemic dynamics and population response in the model calibrated to reporting delay adjusted cases.** **a** Epidemic curve presented per calendar week, contrasted with the model mean and the 95% PI. **b** Posterior distribution of epidemic parameters. **c** Proportion of the population that became infected, diagnosed, or immunized (either through infection or vaccination) throughout the simulation, as well as the proportion that reduced their contacts at any point during the study period.

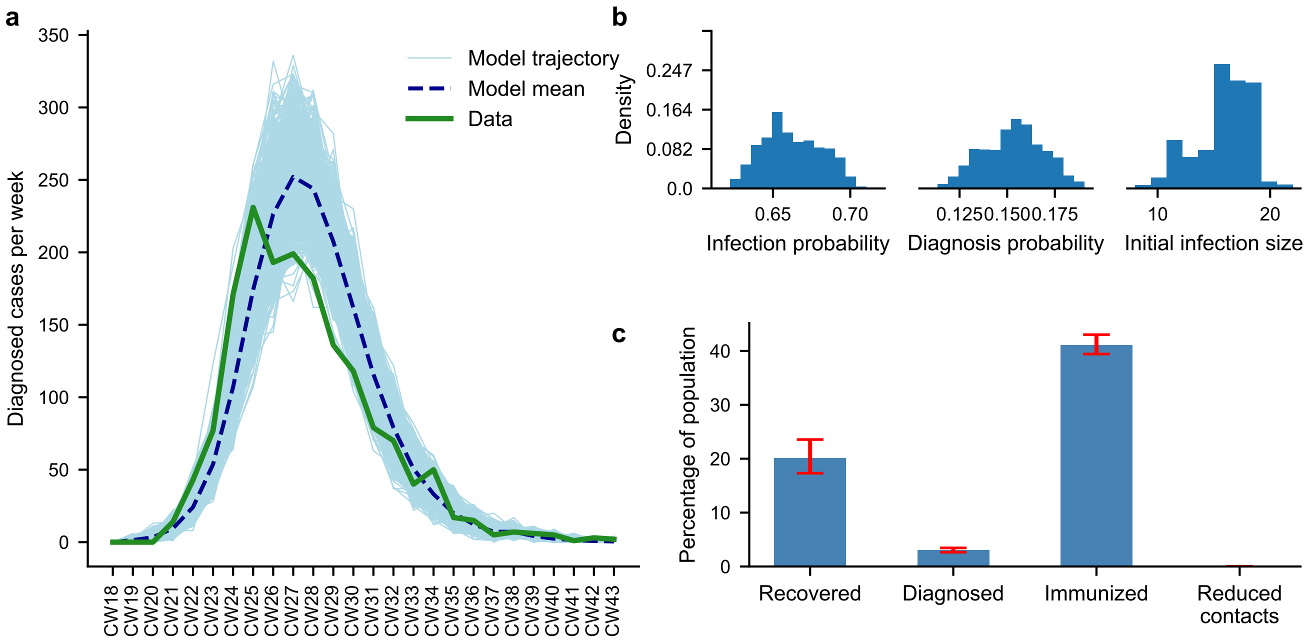

Supplementary Figure 10: **Overview of epidemic dynamics and population response in the model without behavior change.** **a** Epidemic curve presented per calendar week, contrasted with the model mean and the 95% PI. **b** Posterior distribution of epidemic parameters. **c** Proportion of the population that became infected, diagnosed, or immunized (either through infection or vaccination) throughout the simulation, as well as the proportion that reduced their contacts at any point during the study period.

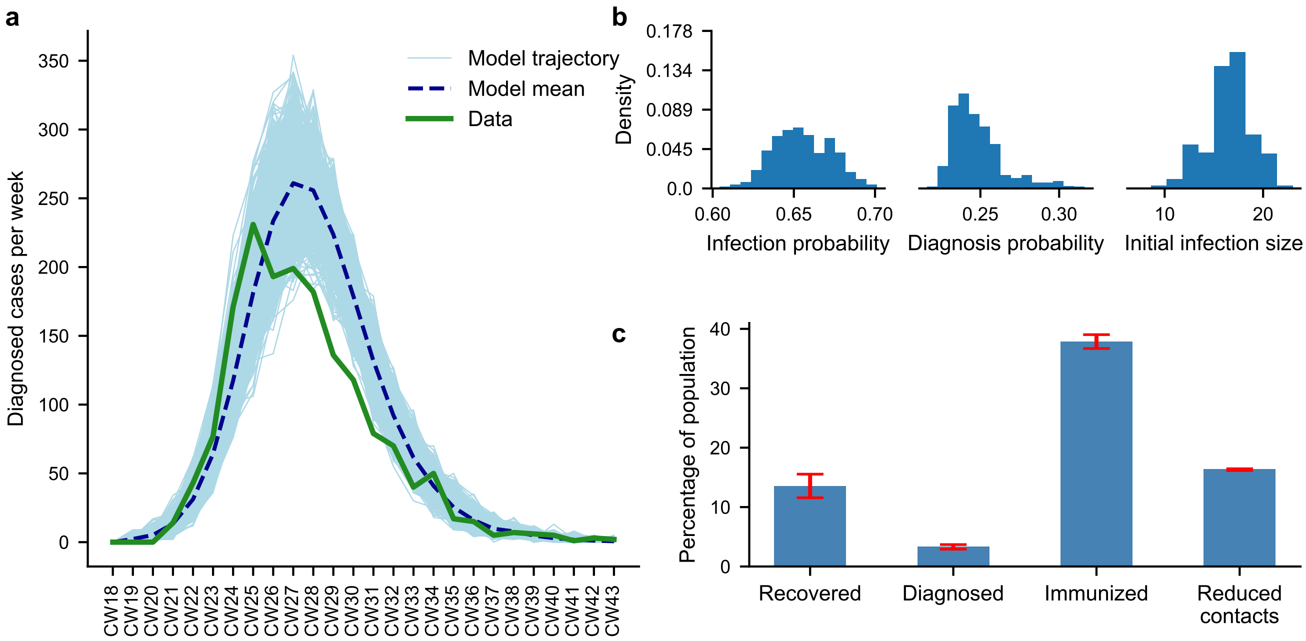

Supplementary Figure 11: **Overview of epidemic dynamics and population response in the model with fewer behavior changes.** **a** Epidemic curve presented per calendar week, contrasted with the model mean and the 95% PI. **b** Posterior distribution of epidemic parameters. **c** Proportion of the population that became infected, diagnosed, or immunized (either through infection or vaccination) throughout the simulation, as well as the proportion that reduced their contacts at any point during the study period.

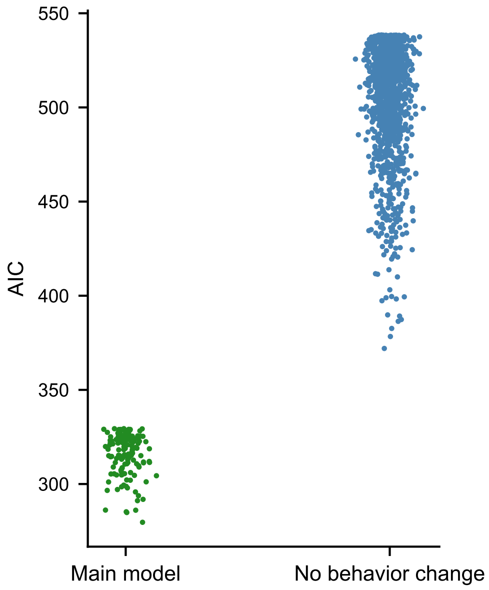

Supplementary Figure 12: Comparison of AIC values for all selected trajectories of the main model and the alternative model without behavior change.

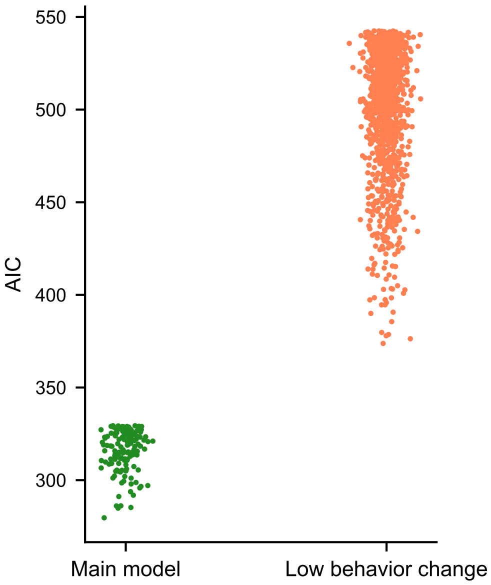

Supplementary Figure 13: **Comparison of AIC values for all selected trajectories of the main model and the alternative model with fewer behavior changes.**

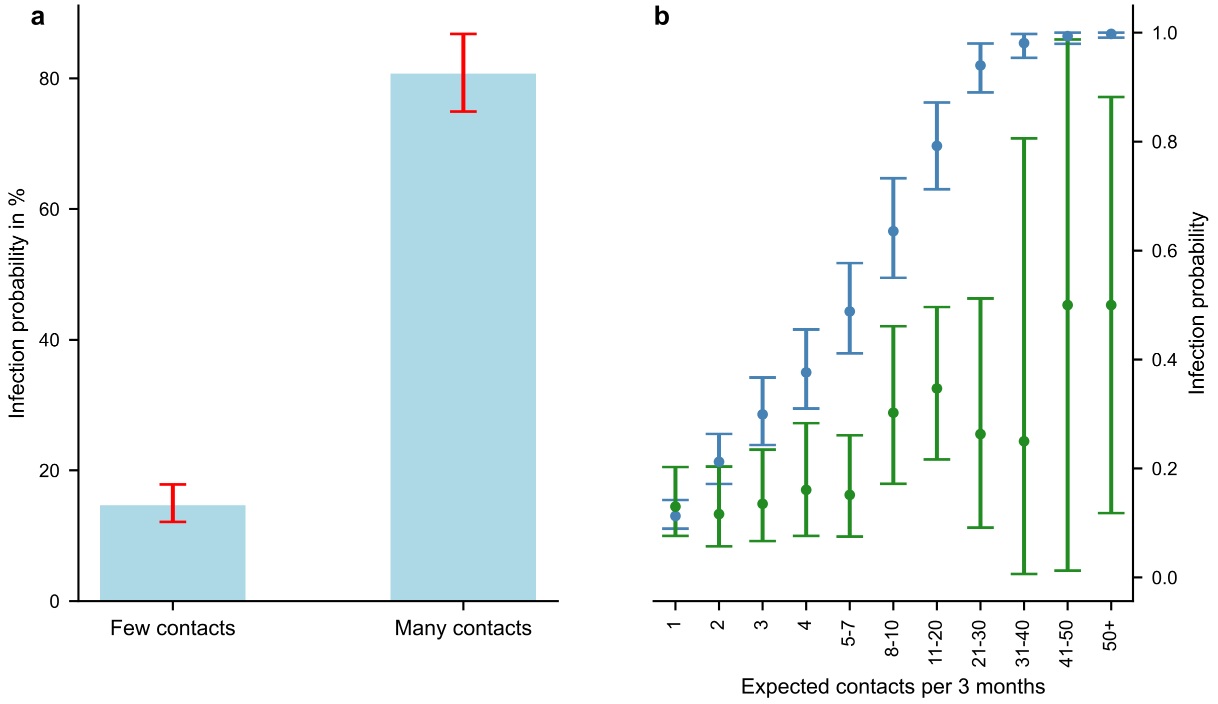

Supplementary Figure 14: **Infection probability per degree category compared to seroprevalence study a** The infection probability with 95% PI for agents with few contacts (less than two per three weeks) and many contacts (two or more per three weeks). **b** The infection probability with 95% PI for contact categories compared to the results of the seroprevalence study [2].

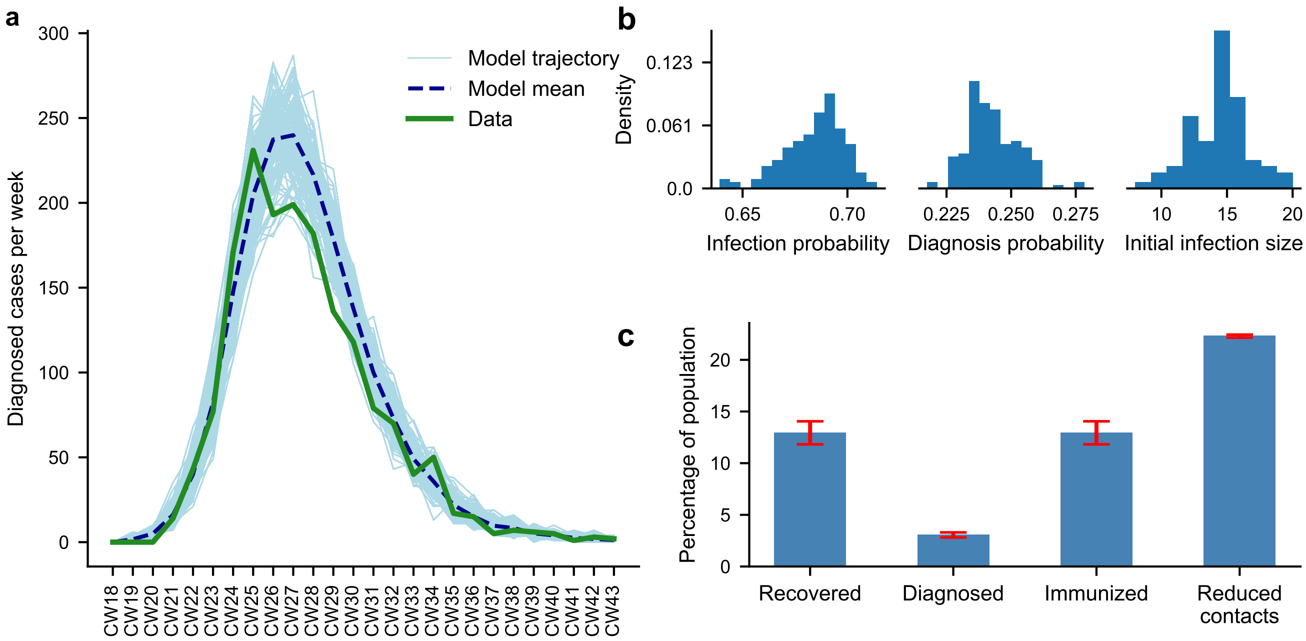

Supplementary Figure 15: **Overview of epidemic dynamics and population response in model without vaccinations. a** Epidemic curve presented per calendar week, contrasted with the model mean and the 95% PI. **b** Posterior distribution of epidemic parameters. **c** Proportion of the population that became infected, diagnosed, or immunized (either through infection or vaccination) throughout the simulation, as well as the proportion that reduced their contacts at any point during the study period.

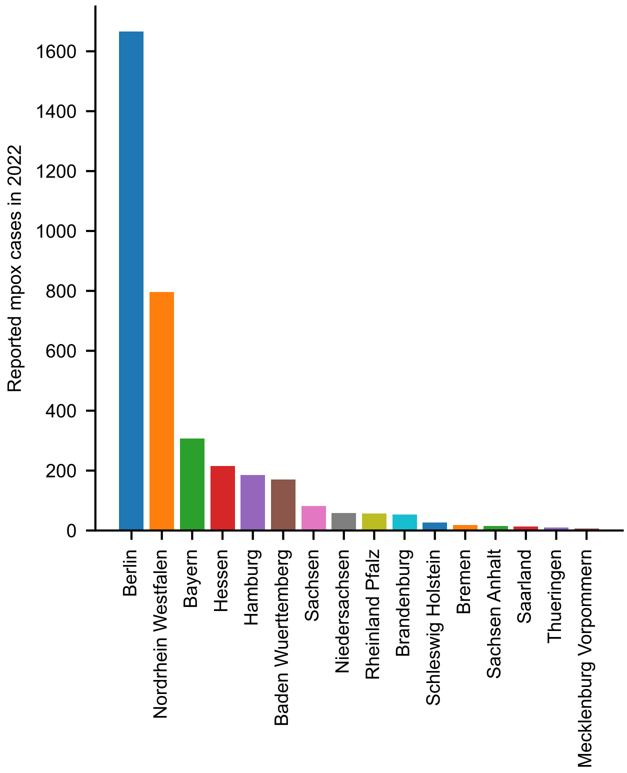

Supplementary Figure 16: Registered mpox cases in 2022 in Germany by federal state.

References

[1] Marta Bertran, Nick Andrews, Chloe Davison, Bennet Dugbazah, Jacob Boateng, Rachel Lunt, Joanne Hardstaff, Melanie Green, Paula Blomquist, Charlie Turner, et al. Effectiveness of one dose of mva–bn smallpox vaccine against mpox in england using the case-coverage method: an observational study. *The Lancet Infectious Diseases*, 23(7):828–835, 2023.

[2] Ulrich Marcus, Janine Michel, Nikolay Lunchenkov, Denis Beslic, Fridolin Treindl, Rebecca Surtees, Christoph Weber, Axel Baumgarten, Andreas Nitsche, and Daniel Stern. A seroprevalence study indicates a high proportion of clinically undiagnosed mpxv infections in men who have sex with men in berlin, germany. *BMC infectious diseases*, 24(1):1153, 2024.

[3] Mindy M Sampson, Christopher M Polk, Robert T Fairman, Michael E DeWitt, Michael K Leonard, Lisa Davidson, Lewis McCurdy, and Catherine L Passaretti. Monkeypox testing delays: The need for drastic expansion of education and testing for monkeypox virus. *Infection Control & Hospital Epidemiology*, 44(2):348–349, 2023.
